# Novel design for a dynamic ankle foot orthosis with motion feedback used for training in patients with hemiplegic gait: a pilot study

**DOI:** 10.1186/s12984-020-00734-x

**Published:** 2020-08-18

**Authors:** Chih-Chao Hsu, Yin-Kai Huang, Jiunn-Horng Kang, Yi-Feng Ko, Chia-Wei Liu, Fu-Shan Jaw, Shih-Ching Chen

**Affiliations:** 1 Institute of Biomedical Engineering, National Taiwan University, Taipei, Taiwan; 2 Department of Physical Medicine and Rehabilitation, Taipei Medical University Hospital, Taipei, Taiwan; 3 School of Medicine, College of Medicine, Taipei Medical University, No. 250, Wuxing St., Xinyi Dist, Taipei City, 110 Taiwan

**Keywords:** Hemiplegia, Ankle, Gait, Stroke, Orthosis

## Abstract

**Background:**

We designed a novel ankle foot orthosis (AFO), namely, ideal training AFO (IT-AFO), with motion feedback on the hemiparetic lower limb to improve ambulation in individuals with stroke-related hemiplegia. We, therefore sought to compare the kinematic parameters of gait between IT-AFO with and without dynamic control and conventional anterior-type AFO or no AFO.

**Methods:**

Gait parameters were measured using the RehaWatch® system in seven individuals with hemiplegia (mean 51.14 years). The parameters were compared across four conditions: no AFO, conventional anterior AFO, IT-AFO without dynamic control, and IT-AFO with dynamic control, with three trials of a 10-m walk test for each.

**Results:**

The dorsiflexion angle increased during the swing phase when the IT-AFO was worn, and it was larger with dynamic control. These data can confirm drop foot improvement; however, the difference between the parameters with- and without-AFO control conditions was not significant in the swing phase. The IT-AFO with or without dynamic control enhanced the loading response to a greater extent between the hemiparetic and unaffected lower limbs than conventional AFO or no AFO. The duration of the stance phase on the hemiparetic lower limb was also longer when using IT-AFO with and without dynamic control than that when using conventional AFO, which improved asymmetry. User comfort and satisfaction was greater with IT-AFO than with the other conditions.

**Conclusions:**

The IT-AFO with dynamic control improved gait pattern and weight shifting to the hemiparetic lower limb, reducing gait asymmetry. The difference with and without dynamic control of IT-AFO is not statistically significant, and it is limited by sample size. However, this study shows the potential of IT-AFO in applying positive motion feedback with gait training.

**Trial registration:**

Taipei Medical University-Joint Institutional Review Board. N201510010. Registered 12 February 2015. http://ohr.tmu.edu.tw/main.php.

## Background

An ankle foot orthosis (AFO) is a commonly used device to improve gait in patients with stroke-related hemiplegia [1]. An AFO provides physical support to the ankle joint and foot [2], with the aim of improving weight-bearing on the affected lower limb. It is estimated that over 4 million people in the United States use an AFO for gait-related impairments [3]. The American Board for Certification in Orthotics, Prosthetics and Pedorthics, Inc. (ABC) reported that in 2016, 74.2% of orthotists’ time was spent in fabricating lower limb orthoses, with AFOs accounting for 36% of these devices. Similarly, in 2014, the Social and Family Affairs Administration in Taipei reported that the highest proportion of subsidies were for AFOs [4].

Stroke is the most common indication for AFO prescription. A stroke is defined as the death of brain cells caused by cerebral ischemia, which results in a wide range of motor impairments, including gait impairment [5]. The prevalence of stroke is approximately 19.3 per 1000 people aged over 35 years in Taiwan [6]. In the United Sates, it is estimated that 795,000 people sustain a new or recurrent stroke every year [7]. Gait function is often affected in stroke survivors [8–10], with AFOs recommended to improve the position of the foot and ankle during the gait cycle [11]. A retrospective analysis concluded that the prevalence rate of AFO use after a stroke was 30.7% in Japan in 2015, with a better Functional Independence Measure score at discharge among patients who were prescribed an AFO than that in patients who did not use an AFO for gait retraining [12].

Conventional AFOs are used to restrict ankle plantarflexion, thus maintaining the hemiparetic foot in a position of dorsiflexion to facilitate swing [13]. However, this restriction in ankle movement disrupts the rhythm of gait and increases energy consumption during walking [14–16]. To alleviate this issue, hinge AFOs were developed to allow some dorsiflexion during the loading response on the affected lower limb, thus slightly reducing the energy cost of hemiparetic gait [17]. Elastic materials (such as carbon fibers) have been included in some AFO designs to provide an assistive function to further reduce energy expenditure [18, 19]. Mechanical features (such as dampers and springs) as well as electronic components (such as magnetorheological braking systems, force and position sensors, accelerometers, and microprocessors) have been included in the hinge to try and improve control over ankle motion [19, 20]. However, to the best of our knowledge, an AFO has not been developed with the specific aim of providing motion feedback for gait training.

Typically, physical therapists use hands-on activities as feedback to facilitate normal movement patterns in conventional gait training [21]. Recently several devices provide “reminding” external feedback for improving gait performance, such as stance-feedback to increase the stance time on the affected side or swing-feedback to decrease the swing time on the affected side [22, 23]. Therefore, we designed an AFO with novel motion feedback mechanism, which is performed by recognition execution of motor learning.

**Fig. 1:**
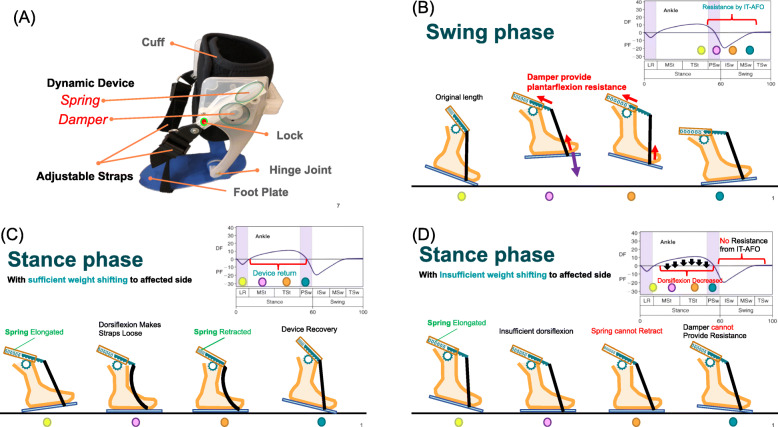
Schematic diagram of the device, including the **a** structure and **b** dynamic components providing plantarflexion resistance in the swing phase, **c** dynamic components restored on the stance phase with sufficient weight shifting to the affected side, and **d** dynamic components not restored in the stance phase with insufficient weight shifting to the affected side

In this study, we describe a novel type of AFO, the ideal training AFO (IT-AFO), which we developed at Taipei Medical University and customized in a patient-specific manner using 3-dimensional (3D) printing for fabrication [24, 25]; it optimizes the alignment of the hinge with the axis of motion of the ankle in the sagittal plane. The IT-AFO includes a dynamic component designed specifically to provide motion feedback during walking. This mechanism design is based on changes in the ankle angle during the gait cycle [26]. There are two dynamic components on both sides of one IT-AFO, and each dynamic component contains a one-way damper (1 Ns/m) and a spring (0.625 kgf), shown in Fig. 1a. Springs are only used to restore components while dampers provide the main plantarflexion resistance. However, springs provide very little plantarflexion resistance during the swing phase (Fig. 1b). Springs can retract the straps when in the stance phase with sufficient weight shifting to the affected side resulting in component restoration (Fig. 1c). Insufficient weight shifting to the affected side decreases the ankle dorsiflexion angle, because straps are still tight when in the stance phase, thus impeding component restoration (Fig. 1d) (straps are still tight because of insufficient ankle dorsiflexion). Users will feel more assisted force on the swing phase after every step with sufficient weight shifting to the affected side on the stance phase. Therefore, IT-AFO has potential for enhancing motor control recognition schema with gait training.

This study aimed to compare the gait kinematics in individuals with stroke-related hemiplegia using IT-AFO with dynamic control, IT-AFO without dynamic control, conventional AFO, and no AFO.

## Methods

Potential participants with stroke-related hemiplegia were recruited from Taipei Medical University Hospital. Patients with lower limb amputation or orthopedic conditions causing deformities affecting gait, open lower limb skin wounds, and those with cognitive deficits that affect the ability to provide consent and follow instructions were excluded. Nine participants (two women and seven men) were recruited. Of these, one man was unable to complete the entire study protocol, and his data were withdrawn from the analysis and another was unable to complete the study; both men were excluded. Therefore, the analysis included seven participants, 29 to 83 years of age (mean, 51.14 years; standard error of the mean [SEM], 7.0). All patients provided informed consent, and the study protocol was approved by the Ethics Committee of the Office of Human Research of Taipei Medical University (N201510010).

All participants had a recovered functional ambulation classification (FAC) [27, 28] score above level IV after their stroke on using conventional ankle foot orthoses on the hemiparetic side. Basic clinical data, including the manual muscle testing score (higher score indicative of greater strength) [29], the modified Ashworth Scale score (lower score indicative of more normal muscle tone/spasticity) [30], and the Berg Balance test score (higher score indicative of greater balance function) [31] were collected before the trial, as shown in Additional File 1.

For 3D printing, a portable 3D Structure Sensor [32] was used to capture the contours of the areas of the hemiparetic lower limb that would be covered by the AFO, at a scanning frequency of 30 frames/s. The high-resolution image file (OBJ format) was transferred by email. All scans were obtained with the patients lying supine, and the target leg was supported by a tripod placed outside the bed.

The OBJ file was converted to the STL format and used for 3D printing of the AFO, using a custom program developed at the Taipei Medical University. For 3D printing, the required anatomical landmarks (outer edges of the first and fifth metatarsophalangeal joints, bottom edges of the second to third metatarsophalangeal joints, lateral malleolus and medial malleolus, upper back edge of calcaneus) were digitized on the 3D graphical images, with all components of the process (cutting, meshing, and customization) performed automatically. Of note, several details could be adjusted manually before 3D manufacturing of the AFO.

All AFOs were printed using an Ultimaker 3D printer (Ultimaking Ltd., 4191PL Geldermalsen, Netherlands) [33], with 4611 Nylon (Zig Sheng Industrial CO., Ltd) used for all AFOs. This material has good wear and abrasion resistance characteristics with a general thickness of 3 mm. Fabrication of the AFO using 3D printing allowed us to align the axis of motion of the orthosis with the sagittal plane of motion of the ankle joint as closely as possible. The motion-controlled straps on the IT-AFO provided precise control of the position of the metatarsophalangeal joints and optimal control of ankle motion, both of which are important to avoid excessive spastic response.

**Fig. 2:**
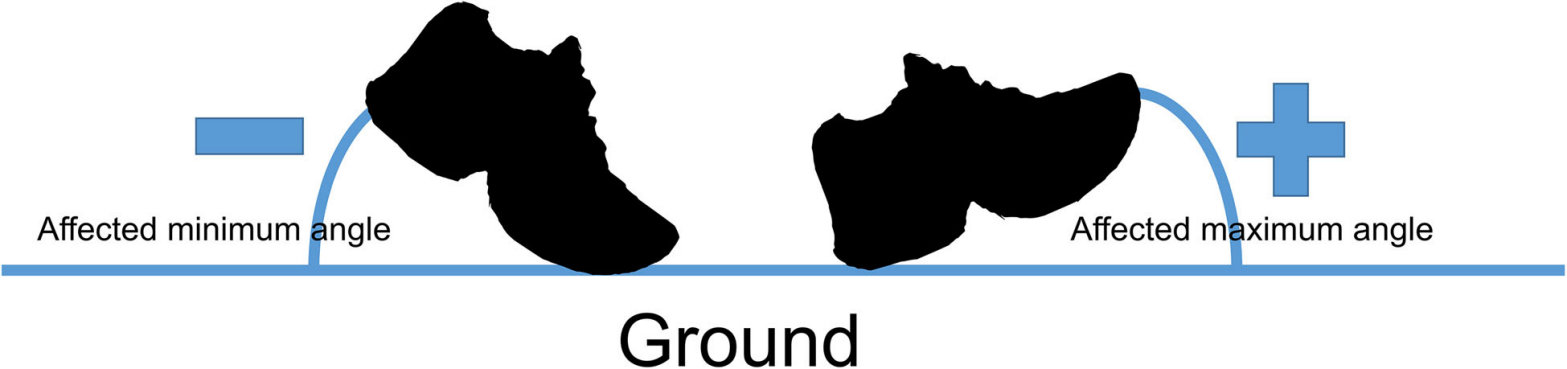
Schematic representation of the minimal and maximal angles of the foot at toe-off and heel-strike, respectively, on the affected side

The RehaWatch system (RehaWatch® system; HASOMED® GmbH, Magdeburg, Germany) was used to detect gait parameters during a 10-m walk test. The sensors (Analog Devices, Norwood, MA, USA) were placed below the lateral malleolus. Each sensor contained three accelerometers (dynamic range, ±5 g) and three gyroscopes (dynamic range, ±600°/s) to measure foot motion [34] across the events of the gait cycle (heel-strike, foot flat, and toe-off) and to capture the 6 degrees of freedom kinematics of the gait cycle at a sampling rate of 512 Hz. The minimum foot angle (relative to the ground) at toe-off and maximum foot angle at heel-strike were calculated (Fig. 2) for both the hemiparetic and unaffected sides. Using the RehaWatch system, the minimal angle corresponds to the angle of the ankle plantarflexion during the pre-swing phase of gait, and the maximal angle corresponds to the angle of ankle dorsiflexion at initial contact (heel-strike) From the heel-strike and toe-off captured bilaterally, the kinematic parameters of the gait cycle could be calculated automatically: stride length, foot height, and walking speed. The calculated gait kinematics were referenced to the normal distribution of values for the RehaWatch system, which were derived from a population of 1860 healthy individuals aged between 5 and 100 years [35]. Measures were obtained for walking conditions: without an AFO, wearing an anterior-type of AFO, wearing the IT-AFO without dynamic control, and wearing the IT-AFO with dynamic control.

Every dynamic component contained a lock which could be used to block the dynamic control from the dynamic component as shown in Fig. 1a. When it is locked, the ankle was controlled by material and structure, that two firm stems provided strong resistance force in the sagittal plane. The cross-straps not only transfer the sagittal resistance force form stems, but also control the ankle motion in the frontal plane. Participations were asked to sit and place the affected foot on a wedge, which made the foot maintain dorsiflexion at 5°. Then, the straps are shut tight with the device locked. All controlling force was transferred though the two straps to the metatarsophalangeal joints. IT-AFO with this mode can assist walking as that with conventional AFO function. During lock opening, damping becomes the major provider of resistance force, which allows more ankle mobility for training purposes with second mode.

All trials were performed at the participant’s comfortable walking speed, with three 10-m tests completed for each condition. The participants did not change their shoes in any of the four conditions to avoid interferences with the results. After each block of three trials for each AFO condition, participants were asked to rate their “comfort” with the device and the “assistance” provided for the gait.

The following gait variables calculated bilaterally were used in the analysis: minimum and maximum angle, loading response (time from the maximum angle to foot flat), and loading score (calculated by combining the loading response for both the affected and unaffected lower limbs and comparing them to the RehaWatch normal references). The mean ± SEM was calculated for each variable. The sphericity of the data was assessed using the Mauchly’s test, with a Greenhouse-Geisser procedure used if the assumption of sphericity was violated to adjust the degrees of freedom to yield a more conservative *F*-ratio. Within-subject differences in gait parameters under the four walking conditions were evaluated using a repeated-measure analysis of variance. A paired sample *t-*test was used for pair-wise comparison of the group means for the four conditions. All analyses were performed using IBM Statistical Package for Social Sciences (SPSS) version 22, with a *p*-value (two-tailed) < 0.05 considered significant.

## Results

**Fig. 3:**
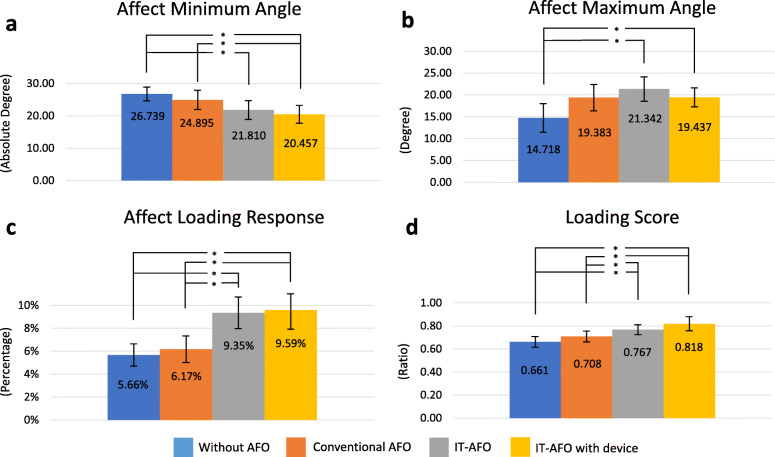
Measured variable of gait on the affected side: **a** minimum angle; **b** maximum angle; **c** loading response; and **d** loading score

Basic clinical data for our study group are shown in Table 1. Three and four participants had right and left hemiplegia, respectively. In addition, six of the seven participants had FAC classification V, with the other having classification IV. The minimum angle (shown in Fig. 3a) was smaller when using the IT-AFO with dynamic control (20.46 ± 2.77°) than that under the conventional AFO (24.90 ± 2.95°) or no AFO (26.74 ± 2.13°) conditions. The minimum angle was smaller for the IT-AFO without dynamic control (21.81 ± 2.89°) than that without an AFO. Similarly, the maximum angle was larger for both the IT-AFO with dynamic control (19.44 ± 2.18°) and that without dynamic control (21.34 ± 2.81°) than for that without an AFO (14.72 ± 3.27°). However, there was no significant difference in the maximum angle between the two IT-AFO conditions and when using AFO (19.38 ± 3.03°), as shown in Fig. 3b.

**Table 1:** Relevant information on participants

No.	Sex	Age (years)	Affected side	Date of onset (months)	Berg Balance test	FA	Diagnosis
1	Female	55	R	2	52	V	Pontine lacunar infarction
2	Male	29	L	62	54	V	Parietal lobe ICH.
3	Male	67	L	25	43	IV	MCA infarction
4	Female	41	R	78	45	V	High frontoparietal region ICH
5	Male	41	L	51	48	V	Putamen ICH
6	Male	83	R	34	50	V	Corona radiate lacunar infarction
7	Male	42	L	21	51	V	Thalamic ICH
(Mean ± SEM)	Sex	51.14 ± 7.00		38.86 ± 9.92	49.00 ± 1.4		

*MCA* middle cerebral artery, *ICH* intra-cerebral hemorrhage, *SEM* standard error of the mean, *FAC* functional ambulation classification

Although the walking speed increased slightly with conventional AFO, IT-AFO, and with the device (0.465 ± 0.101 m/s, 0.463 ± 0.095 m/s and 0.453 ± 0.076 m/s), the difference was not statistically significant (without-AFO 0.431 ± 0.081 m/s). The double support of the affected side was higher for the IT-AFO without dynamic control (17.6 ± 3.6%) and with dynamic control (19.2 ± 3.7%) than that for the conventional AFO (15.1 ± 3.2%) and no AFO (15.7 ± 2.1%) conditions, as shown in Additional File 2. The loading response and loading score were higher for the IT-AFO without dynamic control (9.35 ± 1.37% and 0.767 ± 0.043, respectively) and with dynamic control (9.59 ± 1.69% and 0.818 ± 0.061, respectively) than those for the conventional AFO (6.17 ± 1.15% and 0.708 ± 0.047, respectively) and no AFO (5.66 ± 0.97% and 0.661 ± 0.046, respectively) conditions, as shown in Fig. 3c. These differences between conditions were retained when using the norm-referenced loading response for individuals of the same height and age (Fig. 3d).

**Fig. 4:**
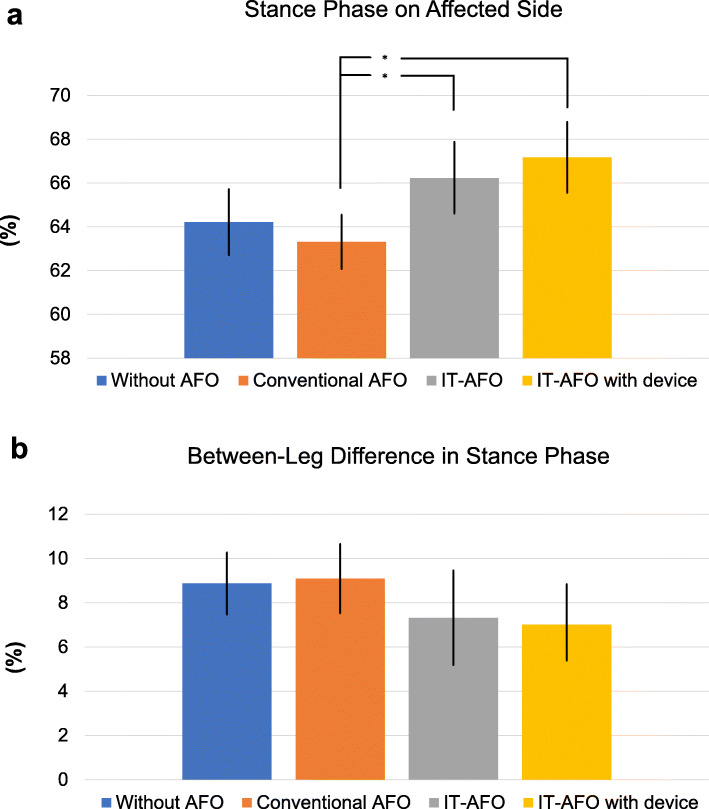
Percentage of the stance phase on the affected side

The stance phase on the hemiparetic lower limb increased significantly when using the IT-AFO without dynamic control (66.23 ± 1.65%) and with dynamic control (67.17 ± 1.62%) than that under the conventional AFO (63.31 ± 1.24%) condition. When wearing a conventional AFO, the stance phase on the hemiparetic limb was lower than that without an AFO (64.21 ± 1.50%), as shown in Fig. 4a. The absolute difference in the duration of the stance phase between the hemiparetic and unaffected lower limbs is shown in Fig. 4b. The between-limb difference was smaller when using the IT-AFO, either with (7.01 ± 1.82%) and without (7.32 ± 2.14%) dynamic control (7.01 ± 1.82%) than that when using the conventional AFO (9.09 ± 1.57%) or no AFO (8.87 ± 1.40%).

**Fig. 5:**
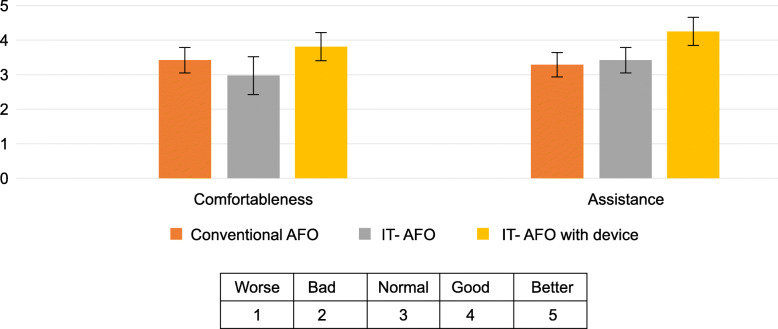
User satisfaction with the device

Participant-reported comfort (C) and assistance (A) were highest for the IT-AFO with dynamic control (C, 3.86 ± 0.40, and A, 4.29 ± 0.36), followed by the IT-AFO without dynamic control (C, 3.00 ± 0.53, and A, 3.43 ± 0.37) and lowest for the conventional AFO (C, 3.43 ± 0.37, and A, 3.29 ± 0.36), as shown in Fig. 5.

## Discussion

Using the RehaWatch system, the minimal angle corresponds to the angle of ankle plantarflexion during the pre-swing phase of gait, and the maximal angle corresponds to the angle of ankle dorsiflexion at initial contact (heel-strike) [36]. Therefore, the IT-AFO increased ankle dorsiflexion during pre-swing and initial contact (Fig. 3a and b). The facilitation of ankle dorsiflexion was greater for the IT-AFO with dynamic control than that without, with dynamic control increasing weight shifting to the hemiparetic lower limb and a longer stance phase relative to the condition without dynamic control or when using a conventional AFO (Fig. 4a). These improvements in weight shifting to the hemiparetic lower limb with dynamic control of the IT-AFO improved the bilateral symmetry of gait (Fig. 4b). Moreover, increase in the loading response (Fig. 3c and d) would produce a smoother transfer of weight between the limbs and therefore allow users to achieve better gait pattern more easily [37]. Although the differences in the loading response were not significantly different for the IT-AFO with and without dynamic control, overall, the IT-AFO with dynamic control is deemed to be useful to normalize the gait parameters among patients with a stroke-related hemiplegia.

The design of the dynamic IT-AFO not only aims to normalize the parameters of hemiplegic gait but also enhances gait training through the motion feedback. The dynamic control mechanism depends on the interaction between weight shifting and the ankle angle over the gait cycle. The IT-AFO uses straps to restrict ankle plantarflexion over the entire gait cycle, with the dynamic control providing resistance against plantarflexion during the swing phase, which improves the loading response, as well as the first and second rockers of the foot. Furthermore, as the length of the dynamic device increases during the swing phase, that energy can be recovered during the loading response to control the ankle dorsiflexion and consequently, the anterior motion of the trunk over the foot. Dynamic control recovery depends on the increase in ankle dorsiflexion during the stance phase, and the amount of ankle dorsiflexion is related to the amount of weight shifting to the affected side; therefore, more weight is shifted to the affected side when in the stance phase. During the swing phase and owing to feedback, there is more support for dorsiflexion [26]. Therefore, users will experience an increase in dorsiflexion assisting force, generated by the AFO, which will assist the transition into the next swing phase. Together, these advantages of dynamic control improve the gait pattern in a hemiplegic patient.

Hemiplegic gait is characterized by reduced weight-bearing on the affected side because of insufficient muscle strength, disorderly motor control, and spasticity [38]. The resulting asymmetry increases the individual’s fear of falling, which can result in secondary sarcopenia because of decreased walking, as well as the potential for scoliosis because of persistent asymmetrical stance [39, 40]. Secondary development of knee pain is another common problem, as the patient will tend to use joint hyperextension to control the stance phase to compensate for weakness or excessive ankle plantarflexion, which increase the difficulty in transitioning from stance to swing, therefore causing discontinuities in the gait cycle [41]. Theoretically, gait training using the IT-AFO with dynamic control would improve weight shifting to the affected lower limb by improving the position of the ankle in dorsiflexion during stance and, thus, reduce the potential of developing knee pain by preventing knee hyperextension.

The propose of this study was to prove that IT-AFO with device can provide a feedback that induced patients to shift weight to the affected side during training. IT-AFO are not the ones that participants usually get used to, but the walking speed showed no significant change from that with conventional AFO. Furthermore, participants needed to pay more attention towards ankle control with weight shifting when using the dynamic device; yet, the walking speed had no significant change from that with the conventional one. In contrast, the double support on the affected side increased most when using IT-AFO with device (Additional File 2), corresponding with increased loading response (Fig. 3), which is the index that refers to weight acceptance on the affected side. These results might have been related to participants trying to modify the way of weight shifting when walking with IT-AFO and causing an increase in the stance phase with similar walking speed. However, this pilot study showed some limitations, such as detecting EMG and foot plate pressure for references of muscle activity and weight-bearing for the perfect gait analysis.

In contrast to the IT-AFO with dynamic control, conventional AFOs function primarily to restrict excessive ankle plantarflexion [42] and also inhibit the rockers of the gait cycle of the first and second foot, which shortens the loading response and increases the kinematic asymmetry between the lower limbs, thus increasing the energy demands of walking [26]. Accordingly, newer AFOs have aimed to resist (rather than restrict) ankle plantarflexion [43], which is consistent with the mechanics of the IT-AFO.

Three-dimensional printing fabrication is used for optimizing the alignment of the hinge with the sagittal plane axis of the motion of the ankle and the position of the strap fixation under the metatarsophalangeal joints [24, 25]. The circular structures outside the cuff and 4611 Nylon material provide better flexibility and elasticity, that provide better ankle control to IT-AFO and apply the function of its dynamic components. For example, there is extra dorsiflexion in the pre-swing phase, and the IT-AFO structure could recover immediately before the initial swing phase. Therefore, the dynamic components provide plantarflexion resistance directly on the swing phase.

The limitations of our study need to be acknowledged. Foremost, our sample size was small (seven participants), and all participants had an FAC classification of IV or V. These features of our study groups might explain the absence of significant differences among the groups using IT-AFO with and without dynamic control. Gait parameters were measured for the first time using the IT-AFO. therefore, the effects of the IT-AFO should be examined after practice to confirm the benefit of motor learning for gait training with IT-AFO dynamic control. Lastly, we only evaluated the temporal components of gait; there would be a benefit of conducting full gait analysis, including kinetics and muscle activity profiles.

## Conclusions

Our novel IT-AFO with dynamic control improves the overall kinematics of gait, with a specific benefit of improving the loading response and weight shifting to the hemiparetic lower limb. These effects could be beneficial for enhancing gait training in patients with stroke-related hemiplegia.

## Supplementary information

**Additional file 1.** Scores for manual muscle testing and the modified Ashworth Score. Presents the Manual Muscle Testing score (blue bars) and the Modified Ashworth Scale score (orange bars) for each participant.

**Additional file 2.** Walking speed and percentage of double support on affected side. Presents the average of walking speed and average percentage of double support on the affected side of each condition.

## Data Availability

The datasets supporting the conclusions of this article are available in the International Organization for Standardization. ISO 8549-3:1989 - Prosthetics and orthotics -- Vocabulary -- Part 3: Terms relating to external orthoses. 1989:5. Repository: https://www.iso.org/standard/15802.html. Accessed June 27, 2018; Ministry of Health and Welfare, Taiwan. Statistical analysis report on the service of orthoses service 2014, Social and Family Affairs Administration. 2014. Repository: https://repat.sfaa.gov.tw/files/104年輔具服務彙整分析報告.pdf. Accessed July 17, 2018; Occipital. Specifications of structure sensors. Repository: https://structure.io/support/what-are-the-structure-sensors-technical-specifications. Accessed August 1, 2018.

## Abbreviations

AFO: Ankle foot orthosis
IT-AFO: Ideal training ankle foot orthosis
FAC: Functional ambulation classification
