# Supplementary figures and images for: Novel design for a dynamic ankle foot orthosis with motion feedback used for training in patients with hemiplegic gait: a pilot study
Source: J Neuroeng Rehabil. 2020 Aug 18;17:112. doi: 10.1186/s12984-020-00734-x (PMC7433152; doi:10.1186/s12984-020-00734-x)

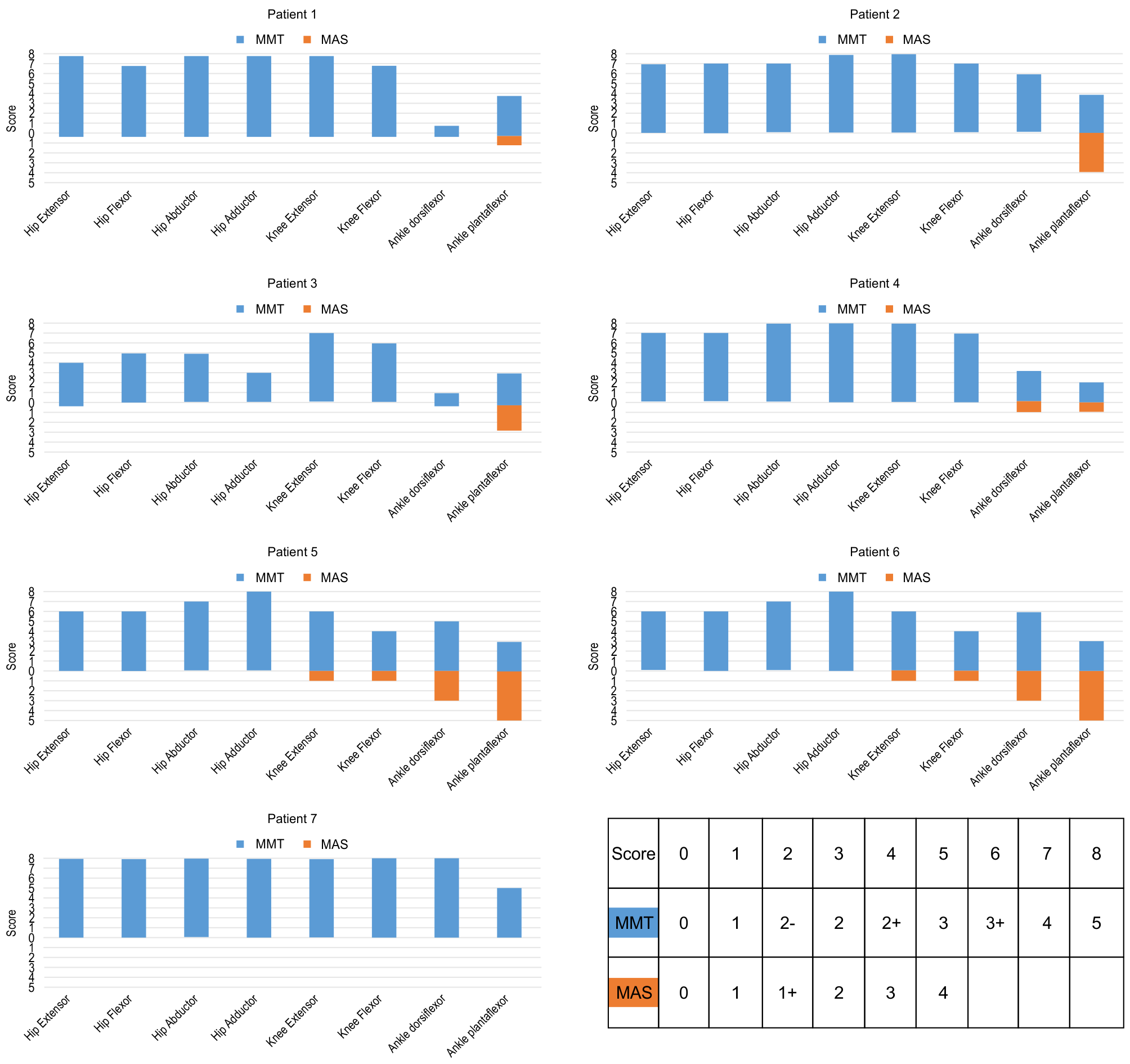

Supplement: Supplementary file 1 — Additional file 1. Scores for manual muscle testing and the modified Ashworth Score. Presents the Manual Muscle Testing score (blue bars) and the Modified Ashworth Scale score (orange bars) for each participant. [file 12984_2020_734_MOESM1_ESM.tif]

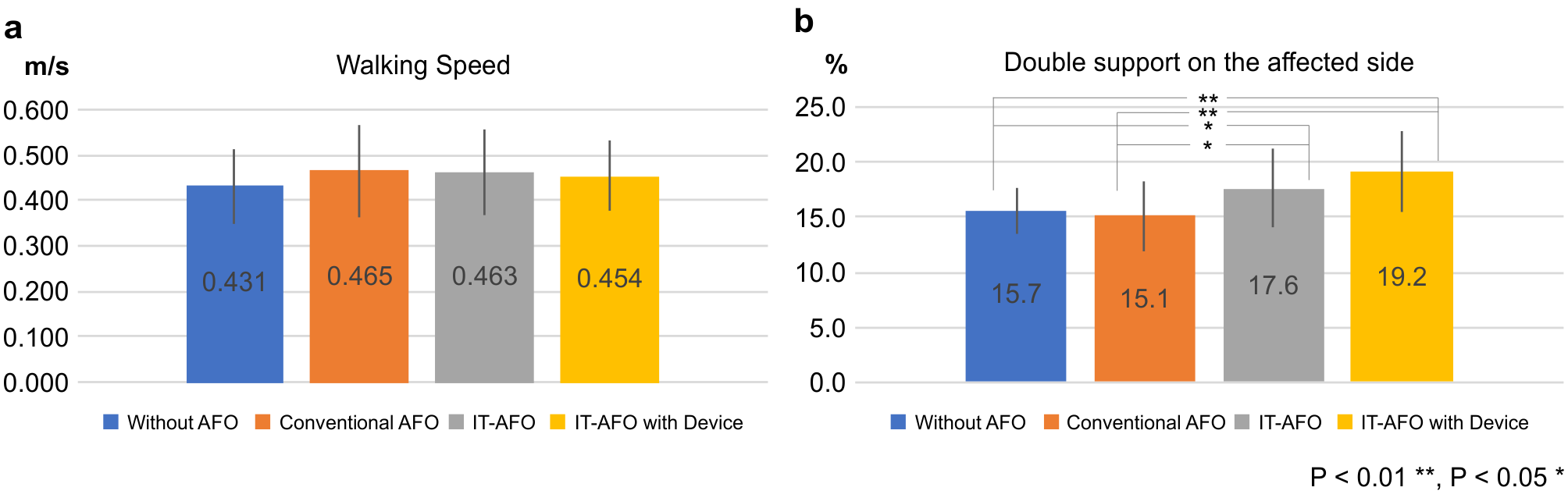

Supplement: Supplementary file 2 — Additional file 2. Walking speed and percentage of double support on affected side. Presents the average of walking speed and average percentage of double support on the affected side of each condition. [file 12984_2020_734_MOESM2_ESM.tif]
